# Knee Flexion and Popliteal Artery Contrast Arrival in Peripheral Arterial Disease: A Pilot Analysis of Bolus Tracking Monitoring Data

**DOI:** 10.3400/avd.oa.26-00095

**Published:** 2026-10-20

**Authors:** Masayuki Tokunaga, Masamitsu Suhara, Hiroyuki Nagita, Hiroyuki Saigusa, Keitaro Sakai, Masaki Okada, Yuichi Suzuki, Shiori Amemiya, Toshihiro Hayashi, Katsuyuki Hoshina, Osamu Abe

**Affiliations:** 1 Department of Radiology, The University of Tokyo Hospital, Tokyo, Japan; 2 Department of Vascular Surgery, The University of Tokyo Hospital, Tokyo, Japan; 3 Department of Peripheral Vascular Surgery, Sakakibara Heart Institute, Tokyo, Japan; 4 Department of Vascular Surgery, International University of Health and Welfare, Mita Hospital, Tokyo, Japan

**Keywords:** peripheral arterial disease, popliteal artery, knee flexion, CT angiography, bolus tracking

## Abstract

**Objectives:**

Peripheral arterial disease (PAD) symptoms can worsen with knee flexion. We examined whether time–enhancement curve (TEC) analysis of discarded bolus tracking data could detect positional differences in popliteal arrival.

**Methods:**

In this secondary analysis of a dedicated research protocol (not routine practice), 22 patients with PAD underwent bilateral lower-extremity CT angiography in extended and flexed positions, at ≈90-degree and at maximum flexion. Arrival time, rise rate, and initial area under the curve (iAUC) were extracted. The flexed and extended limbs were compared with Wilcoxon signed-rank tests.

**Results:**

At maximum flexion, the flexed limb showed a median arrival delay of +1.5 s (p = 0.0002); the within-subject increase from 90-degree flexion to maximum flexion was significant (+0.5 s; p = 0.018). Exploratory subgroup analysis showed significant delays at both angles in unilateral (contralateral ankle–brachial index ≥0.9; n = 15) but not bilateral PAD (n = 7). The rise rate and iAUC did not differ.

**Conclusions:**

The flexed (affected) limb showed delayed popliteal arrival, which was greater at maximum flexion. Because limb position and disease severity were confounded by design, this association is not evidence of causation. These preliminary findings support the feasibility of bolus tracking–derived TEC analysis and motivate within-limb repeated-measures studies.

## Introduction

Peripheral arterial disease (PAD) affects over 230 million people worldwide, with prevalence increasing substantially with age.^1–3)^ The popliteal artery, which traverses the posterior knee joint, is particularly vulnerable to mechanical stress during knee flexion due to its anatomical course through the popliteal fossa.^4,5)^ Sustained knee flexion is common in daily life—for example, during kneeling, squatting, stair climbing, prolonged sitting in vehicles, and the formal seated posture (seiza) customary in Japan—so any position-dependent change in inflow is potentially relevant to everyday activities in patients with PAD. Recent morphological studies have characterized vessel elongation, cross-sectional area reduction, and kink formation in the popliteal artery during knee flexion.^6,7)^ Notably, Nagita et al. recently used three-dimensional computed tomography reconstruction and computational fluid dynamics to demonstrate that highly flexed knee positions can produce kinks in the popliteal artery that create an unfavorable computed hemodynamic state.^6)^ However, these computational predictions have not been complemented by direct in vivo functional measurements in patients with PAD, and the functional consequences of positional changes in this population remain incompletely characterized.

Time–enhancement curve (TEC) analysis provides a method for evaluating contrast medium dynamics within blood vessels.^8–10)^ The fundamental principle relies on tracking temporal changes in CT attenuation following contrast injection, thereby enabling assessment of blood flow characteristics without requiring specialized perfusion imaging protocols.^11–13)^ Previous studies have demonstrated the utility of dynamic CT perfusion for assessing lower-extremity blood flow,^14,15)^ but these techniques require dedicated acquisition protocols, with associated increases in radiation exposure and examination time.

Bolus tracking, routinely employed during CT angiography to optimize contrast timing, generates serial low-dose monitoring images that capture contrast arrival at a specified anatomical location.^16,17)^ These monitoring data, which are typically discarded after triggering the diagnostic acquisition, contain temporal information regarding contrast dynamics that could potentially be repurposed for hemodynamic assessment. While Doppler ultrasound remains the primary modality for hemodynamic assessment of peripheral arteries,^18,19)^ it is operator-dependent and cannot simultaneously evaluate bilateral limbs under identical conditions. A CT-based approach using already-acquired monitoring data could provide complementary, reproducible measurements without additional image acquisition.

Building on the morphological and computational findings of Nagita et al.,^6)^ the present study aimed to examine whether TEC parameters derived from bolus tracking monitoring data could detect positional differences in popliteal contrast arrival during knee flexion. Given the study design, in which the affected limb was consistently assigned to the flexed position, we framed this work as a methodological pilot investigation rather than a definitive test of flexion-induced flow impairment. Because the affected limb was, by design, always the flexed limb, a single between-limb comparison cannot separate positional from disease-related effects. We therefore also examined the within-limb change in arrival delay between 90-degree flexion and maximum flexion, in which each limb’s fixed disease burden cancels out and which is consequently less susceptible to baseline interlimb asymmetry. We hypothesized that, if the bolus tracking-based TEC approach was sensitive to positional hemodynamic changes, contrast arrival in the flexed limb would be delayed relative to that in the extended limb, with a greater effect at higher flexion angles.

## Materials and Methods

### Study design and population

This study was a secondary analysis of a prospective cohort study investigating morphological and hemodynamic changes in the popliteal artery during knee flexion.^6)^ The present study draws on the same patient cohort as the previously published morphological analysis by Nagita et al.^6)^ The 2 studies analyzed different data domains: Nagita et al. used 3-dimensional reconstructions derived from the diagnostic helical CT angiographic acquisitions, whereas the present study uses the serial low-dose monitoring images acquired at 0.5-s intervals during bolus tracking, which are routinely discarded after triggering the diagnostic acquisition. The imaging protocol itself was designed for that prospective study: the knee-flexion positioning, the 2-position acquisition, and the division of the contrast dose between 2 injections were performed specifically for research purposes and do not represent routine clinical lower-extremity CT angiography. What the present analysis repurposes is the bolus tracking monitoring series generated within that protocol, not the protocol as a whole. The original prospective study was approved by the Institutional Research Ethics Committee of The University of Tokyo Hospital (Approval no. 2021196NI-(2)), and written informed consent was obtained from all participants. The present secondary analysis was separately approved by the same committee (Approval no. 11605-(7)), with the requirement for additional informed consent waived due to the retrospective nature of the analysis.

The original cohort comprised 30 patients with lower-extremity arterial disease who underwent CT angiography between November 2021 and April 2023. The original inclusion criteria were as follows: (1) age >20 years; (2) presence of PAD, defined as an ankle–brachial index (ABI) ≤0.90 in the limb designated for flexion (the affected limb)^20,21)^; and (3) ability to undergo CT angiography in both extended and flexed knee positions. The original exclusion criteria were as follows: (1) history of lower-extremity arterial disease treatment for femoropopliteal lesions or artificial joints in the lower limb; (2) decreased renal function (estimated glomerular filtration rate <45 mL/min/1.73 m^2^; patients undergoing dialysis were included); (3) contrast agent allergy or asthma; and (4) inability to bend the knee. For the present TEC analysis, we applied the following additional inclusion criteria: (1) availability of bolus tracking monitoring data from both extended and flexed positions; and (2) adequate image quality permitting reliable region of interest (ROI) placement and TEC parameter extraction. Of the original 30 patients, 22 met these additional criteria and were included in the final analysis.

### CT angiography protocol

All examinations were performed using a 320-multidetector-row CT scanner (Aquilion PRISM Edition; Canon Medical Systems, Tochigi, Japan). Detailed scanning parameters for the diagnostic helical acquisition have been described previously.^6)^ Patients were positioned supine with 1 knee extended and the contralateral knee flexed, thereby enabling bilateral simultaneous imaging. The affected limb (ABI ≤0.90) was placed in a flexed position, supported by a radiolucent cushion to maintain the angle, while the contralateral limb served as the extended control regardless of its ABI value. Patients underwent CT angiography in 2 knee flexion positions: right-angled flexion (approximately 90-degree), which mimicked the sitting position, and maximum flexion, which mimicked the crossed-leg or squatting position. “Maximum flexion” denotes the greatest flexion that each patient could achieve and maintain for the duration of the acquisition under the supine imaging protocol; it is therefore a patient-specific position rather than a standardized physiological angle, and the angle achieved varied between patients (see Results). Imaging in the maximum-flexion position was performed 5 minutes after the right-angled flexion acquisition.

A 22-gauge intravenous catheter was placed in the median cubital vein. The total contrast medium (2 mL/kg body weight, iohexol; Omnipaque 350; GE HealthCare, Chicago, IL, USA) was divided into 2 equal portions and administered at a flow rate of 2.3 mL/s. The first portion (1 mL/kg) was used for the 90-degree flexion acquisition, followed by a 5-minute interval to allow contrast washout, after which the second portion (1 mL/kg) was used for the maximum-flexion acquisition. Bolus tracking monitoring images were acquired at 0.5-s intervals with the following parameters: 120 kVp, 50 mA, and 5-mm slice thickness. Monitoring images were obtained 5 cm above the proximal edge of the patella. Monitoring was initiated 15 s after the start of contrast injection in all cases and continued until the operator confirmed adequate contrast enhancement. Knee flexion angles were measured on multiplanar reformatted images displaying both the femoral and tibial shafts in a single plane and were calculated as the angle between the femoral and tibial midlines. The serial monitoring images were exported for subsequent TEC analysis.

### TEC parameter extraction

ROI placement and measurements were performed using Ziostation2 (Ziosoft, Tokyo, Japan). A circular ROI of approximately 10 mm^2^ was placed within the popliteal artery lumen on each monitoring image, with careful attention to avoid vessel wall and calcified regions. CT attenuation values (Hounsfield units [HU]) were recorded at each time point to construct the TEC (**Fig. 1**). Baseline CT values were defined as the median attenuation of the first 3 monitoring time points (15.0–16.0 s after contrast injection) and were used as the 0 reference (0 HU).

**Fig. 1:**
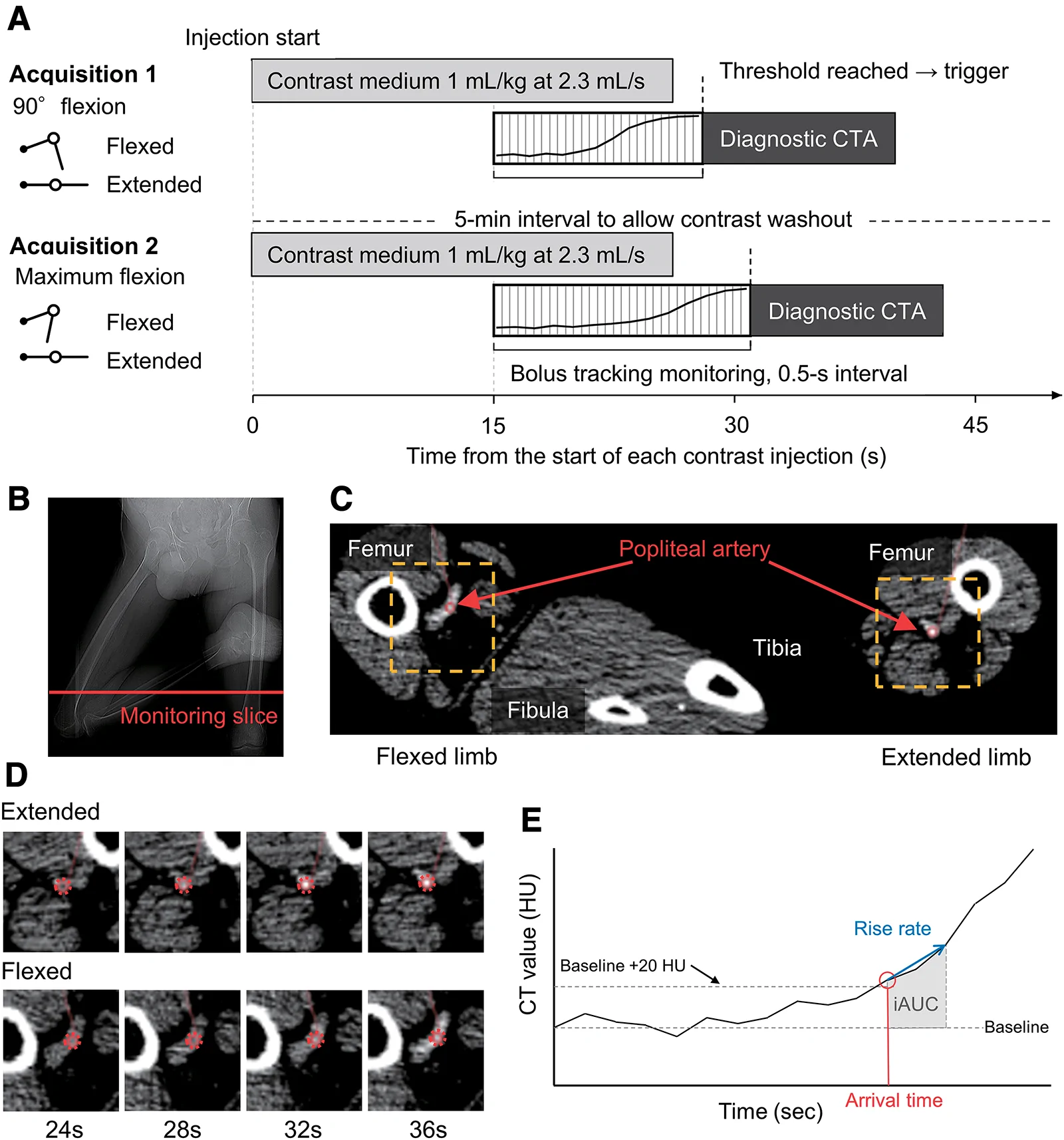
Workflow for TEC analysis of bolus tracking monitoring data. (**A**) The imaging protocol on a common time axis. For each of the 2 acquisitions, the diagram shows the knee position, the contrast injection, the bolus tracking monitoring series, the enhancement threshold that triggered the diagnostic scan, and the diagnostic CT angiographic acquisition. The 2 acquisitions were separated by a 5-minute interval to allow contrast washout. This 2-position protocol was performed specifically for the original research study and does not represent routine clinical CT angiography; only the monitoring series was analyzed in the present work. (**B**) Scout image at maximum flexion. The red line marks the level of the monitoring slice, 5 cm above the proximal edge of the patella. (**C**) Monitoring image of a representative patient at 36 s, showing both limbs in a single slice. Because the target knee is flexed, the slice traverses both the thigh and the calf of that limb. Yellow boxes mark the regions magnified in (**D**). Window level, 100 HU; window width, 200 HU. (**D**) The popliteal artery of each limb at 24, 28, 32, and 36 s after the start of contrast injection (images 19, 27, 35, and 43 of the monitoring series). At each time point, the 2 magnified images are derived from the same monitoring image; therefore, the limbs are shown simultaneously. Each panel covers a 55-mm field of view; circles mark the regions of interest used for measurement (approximately 10 mm^2^; see Materials and Methods). Arrival time was determined from the complete 0.5-s series, not from the frames displayed here. (**E**) Definitions of the derived TEC parameters. Arrival time: the first of 3 consecutive time points exceeding baseline + 20 HU. Rise rate: the maximum slope of the enhancement curve after 3-point median filtering. iAUC: the incremental area under the curve over a 1.5-s window from arrival. TEC, time–enhancement curve; CTA, computed tomography angiography; HU, Hounsfield units; iAUC, initial area under the curve

Three parameters were derived from each TEC:

1. Arrival time: Time from the start of contrast injection to initial contrast arrival, defined as the first of 3 consecutive time points exceeding baseline + 20 HU on the raw data (see **Supplementary Methods** and **Supplementary Fig. S1A**).

2. Rise rate: To reduce high-frequency noise while preserving the temporal characteristics of contrast enhancement, raw TEC data were smoothed with a 3-point median filter.^22)^ The maximum slope of the filtered curve during the upslope phase was calculated as HU/second using a 1-s symmetric window (i.e., from the value 0.5 s before to the value 0.5 s after each time point).

3. Initial area under the curve (iAUC): Integrated enhancement over a 1.5-s window beginning at contrast arrival, calculated from the raw (unfiltered) data to preserve total signal intensity.

For subsequent analysis, arrival time was compared both as an absolute value and as the arrival delay between limbs (flexed minus extended), while the rise rate and iAUC were expressed as ratios (flexed/extended) to account for inter-patient variability in contrast delivery.^23)^

### Justification of the arrival-time threshold

The fixed threshold of baseline + 20 HU was applied uniformly across all datasets rather than tuned per case, thereby removing per-case analyst discretion from the detection step. Across the analyzed datasets, baseline noise (standard deviation, SD) averaged 7.0 HU, so the 20-HU increment corresponded on average to approximately 2.9 SD above baseline (**Supplementary Methods**, **Supplementary Fig. S1A**, **S1B**). To suppress false detection from transient noise excursions, we additionally required 3 consecutive suprathreshold time points (spanning 1.0 s at 0.5-s sampling) on the raw data, ensuring that the detected onset reflected a sustained enhancement rather than a single-frame fluctuation; arrival detection was visually confirmed for every curve. Because a fixed threshold does not scale to the higher baseline noise of the most artifact-affected datasets, this multi-frame requirement and the visual check were the principal safeguards against spurious early detection. Datasets in which the threshold was not exceeded within the available monitoring window were classified as censored rather than excluded, and were addressed in a sensitivity analysis as described below.

### Statistical analysis

Continuous variables are presented as medians with interquartile ranges (IQRs), unless otherwise specified. The primary outcome was the difference in arrival time between the flexed and extended limbs at maximum flexion. Secondary outcomes included the arrival-time comparison at 90-degree flexion, the angle-dependent within-subject change in arrival delay, the rise rate ratio, and the iAUC ratio.

Derivation of the TEC parameters, including 3-point median filtering for the rise-rate calculation, was performed using OriginPro 2026 (OriginLab, Northampton, MA, USA), which was also used for all statistical analyses. Paired comparisons were performed using Wilcoxon signed-rank tests.^24)^ Effect sizes were calculated as r = Z/√n.^25,26)^ Subgroup analyses were performed based on contralateral-limb ABI status (unilateral PAD: contralateral ABI ≥0.9; bilateral PAD: contralateral ABI <0.9). Correlations between continuous variables were assessed using Spearman’s rank correlation coefficient. In datasets in which the arrival threshold was not crossed within the monitoring window (censored cases), arrival time was imputed as the last acquired monitoring time point for the primary Wilcoxon analysis; because true arrival can only be later, this yields a conservative (lower-bound) estimate of the flexed-limb arrival delay. For the secondary ratio outcomes, the rise rate and iAUC of a censored flexed limb, in which no enhancement was detectable within the window, were set to 0. As a censoring-robust sensitivity analysis depending only on the sign of each paired difference, a binomial sign test was also performed. Statistical significance was defined as p <0.05.

## Results

### Patient characteristics

Of the original 30 patients, 8 were excluded due to significant patient motion during monitoring (n = 1), severe beam-hardening artifacts (n = 4), or extensive popliteal artery calcification precluding reliable ROI placement (n = 3). The remaining 22 patients (16 males, 6 females; mean age 73.6 ± 8.0 years) were included in the analysis. Patient demographics and clinical characteristics are summarized in **Table 1**. The mean ABI was 0.68 ± 0.18 in the flexed limbs and 0.91 ± 0.19 in the extended (contralateral) limbs. Fifteen patients had unilateral PAD (contralateral ABI ≥0.9), while 7 had bilateral disease. The mean knee flexion angle was 94.2° ± 6.4° for the 90-degree position and 121.8° ± 13.0° for maximum flexion. A total of 88 TEC datasets were analyzed (22 patients × 2 limbs × 2 flexion angles). Contrast arrival was not detected within the monitoring window in 4 of these 88 datasets (3 at maximum flexion and 1 at 90-degree flexion), all in the flexed limb; these were treated as censored and handled as described above.

**Table 1:** Patient characteristics (n = 22)

Characteristic	Value
Age, years (mean ± SD)	73.6 ± 8.0
Sex, male/female	16/6
Body mass index, kg/m^2^ (mean ± SD)	23.1 ± 3.2
Ethnicity	Asian (100%)
Comorbidities	
Hypertension, n (%)	18 (82)
Diabetes mellitus, n (%)	11 (50)
Dyslipidemia, n (%)	14 (64)
Current smoking, n (%)	6 (27)
Coronary artery disease, n (%)	8 (36)
ABI measurements	
Flexed limb ABI (mean ± SD)	0.68 ± 0.18
Extended limb ABI (mean ± SD)	0.91 ± 0.19
Unilateral PAD, n (%)	15 (68)
Bilateral PAD, n (%)	7 (32)
Knee flexion angles	
90-degree position, degrees (mean ± SD)	94.2 ± 6.4
Maximum flexion, degrees (mean ± SD)	121.8 ± 13.0

ABI, ankle–brachial index; PAD, peripheral arterial disease; SD, standard deviation

### Primary outcome: contrast arrival time

The absolute contrast arrival times and the calculated delays are summarized in **Table 2** (Panel A). In the overall cohort (n = 22), at 90-degree flexion, the median arrival time was 23.5 s (IQR 21.4–29.1) in the extended limb and 27.0 s (IQR 21.4–30.9) in the flexed limb. The resulting median delay was +1.0 s (IQR −1.0 to 3.1), which did not reach statistical significance (p = 0.086; r = 0.37).

**Table 2:** TEC parameters at 90-degree flexion and maximum knee flexion

Panel A. Arrival time (primary outcome)—all patients (n = 22)							
	90-degree flexion				Maximum flexion		
	Extended		Flexed		Extended		Flexed
Arrival time, s	23.5 (21.4–29.1)		27.0 (21.4–30.9)		23.0 (19.9–27.1)		26.3 (21.0–30.4)
	90-degree			Maximum			90-degree vs. Max*
Arrival delay, s	1.0 (−1.0–3.1)			1.5 (0.5–4.5)			0.5 (0.0–2.5)
p-Value	0.086			0.0002			0.018
Effect size (r)	0.37			0.79			—
Panel B. Secondary outcomes—all patients (n = 22)							
	90-degree				Maximum		
	Median (Q1–Q3)		p-Value		Median (Q1–Q3)		p-Value
Rise rate ratio	0.67 (0.41–1.63)		0.23		0.40 (0.01–1.53)		0.15
iAUC ratio	0.91 (0.75–1.08)		0.10		0.93 (0.67–1.23)		0.11
Panel C. Subgroup analysis by contralateral limb ABI status							
	Unilateral PAD (n = 15)				Bilateral PAD (n = 7)		
	90-degree		Maximum		90-degree		Maximum
Arrival time (extended), s	23.0 (21.5–28.0)		22.5 (20.0–27.0)		29.0 (18.5–33.5)		24.0 (18.0–30.5)
Arrival time (flexed), s	28.0 (22.0–29.5)		25.5 (22.0–29.5)		23.5 (18.0–32.5)		27.5 (20.5–34.5)
Arrival delay, s	1.5 (0.0–4.5)		2.0 (0.5–4.5)		−1.0 (−2.5–1.0)		0.5 (0.0–3.0)
p-Value	0.012		0.002		0.50		0.09

*Comparison of arrival delay between 90-degree flexion and maximum flexion.

Values are medians (Q1–Q3). Unilateral PAD: contralateral ABI ≥0.9; bilateral PAD: contralateral ABI <0.9. Arrival delay = arrival time of flexed limb minus arrival time of extended limb. Panel A: p-Values for arrival time and arrival delay: Wilcoxon signed-rank test (flexed vs. extended limb). Panel B: p-Values for secondary outcomes: Wilcoxon signed-rank test against a ratio of 1.0. Panel C: p-Values: Wilcoxon signed-rank test (flexed vs. extended limb).

TEC, time–enhancement curve; PAD, peripheral arterial disease; ABI, ankle–brachial index

At maximum flexion, the median arrival time was 23.0 s (IQR 19.9–27.1) in the extended limb and 26.3 s (IQR 21.0–30.4) in the flexed limb. The median delay significantly increased to +1.5 s (IQR 0.5–4.5), confirming a significant delay in the flexed limb compared to the extended limb (p = 0.0002; r = 0.79) (**Fig. 2A**). As a censoring-robust sensitivity analysis, a binomial sign test was concordant with the primary Wilcoxon results: the flexed limb arrived later in 18 of 19 non-tied pairs at maximum flexion (p <0.0001) and in 14 of 21 non-tied pairs at 90-degree flexion (p = 0.19). Representative TEC curves from a patient with PAD are shown in **Supplementary Fig. S2**.

**Fig. 2:**
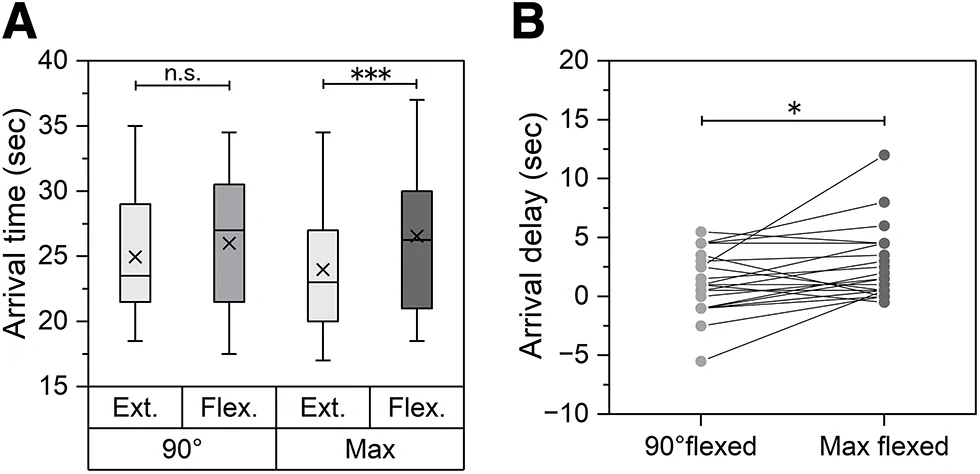
Positional differences in popliteal artery contrast arrival. (**A**) Arrival time in the extended and flexed limbs at 90-degree flexion and maximum knee flexion (n = 22). Box plots show the median (horizontal line), interquartile range (box), and range (whiskers); cross marks indicate mean values. No significant difference was observed at 90-degree flexion (n.s.), whereas maximum flexion produced a significant delay in the flexed limb (***p = 0.0002, Wilcoxon signed-rank test). (**B**) Angle-dependent relationship: each line connects paired measurements from 1 patient, showing the arrival delay (flexed minus extended limb) at 90-degree flexion and maximum flexion. Arrival delay increased significantly from 90-degree flexion to maximum flexion (*p = 0.018, Wilcoxon signed-rank test). Ext., extended limb (internal control); Flex., flexed limb (affected side)

### Angle-dependent relationship

Within-patient comparisons between flexion angles revealed a significant angle-dependent pattern, with greater arrival delays observed at maximum flexion compared to 90-degree flexion (median increase, +0.5 s; IQR, 0.0–2.5; p = 0.018) (**Table 2** Panel A; **Fig. 2B**). This finding is consistent with a mechanical contribution to the observed differences, although the magnitude of the increase equals a single sampling interval and is therefore interpreted with caution (see Discussion).

### Secondary outcomes: rise rate and iAUC

The rise rate ratio (flexed/extended) showed no significant difference from unity at either 90-degree flexion (median 0.67; p = 0.23) or maximum flexion (median 0.40; p = 0.15). Similarly, the iAUC ratio demonstrated no significant changes (90-degree: median 0.91, p = 0.10; maximum: median 0.93, p = 0.11). These parameters exhibited high inter-patient variability (**Table 2** Panel B, **Supplementary Fig. S3**).

### Subgroup analysis

An exploratory subgroup analysis based on contralateral-limb PAD status revealed distinct patterns (**Table 2** Panel C, **Fig. 3**). In patients with unilateral PAD (n = 15), significant arrival delays were observed at both 90-degree flexion (p = 0.012) and maximum flexion (p = 0.002). In contrast, patients with bilateral PAD (n = 7) showed no significant differences at either flexion angle (p = 0.50 and p = 0.09, respectively).

**Fig. 3:**
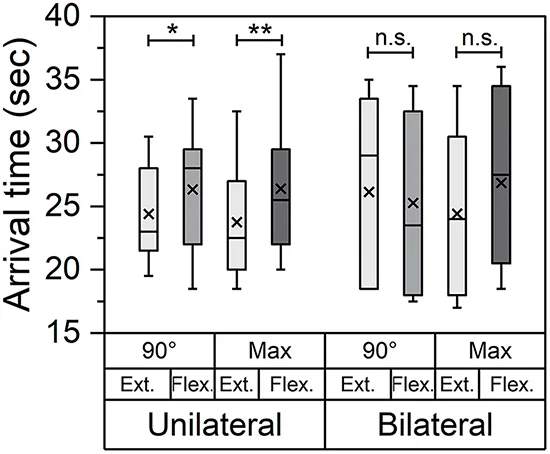
Subgroup analysis comparing unilateral PAD (contralateral ABI ≥0.9, n = 15) and bilateral PAD (contralateral ABI <0.9, n = 7). In patients with unilateral PAD, significant arrival delays were observed at both 90-degree flexion (*p = 0.012) and maximum flexion (**p = 0.002). In patients with bilateral PAD, no significant differences were detected at either flexion angle (n.s.). Box-plot notation is as described in **Fig. 2A**. Ext., extended limb; Flex., flexed limb; PAD, peripheral arterial disease; ABI, ankle–brachial index

### Correlation analysis

Among all patients at 90-degree flexion, the ABI difference (extended minus flexed) showed a moderate positive correlation with arrival time delay (Spearman ρ = 0.49; p = 0.020) (**Supplementary Fig. S4A**), indicating that patients with greater disparity in disease severity between limbs demonstrated larger measurable delays. Within the unilateral PAD subgroup at maximum flexion, flexed-limb ABI showed a moderate negative correlation with arrival delay (ρ = −0.52; p = 0.049) (**Supplementary Fig. S4B**), suggesting that more severe PAD was associated with a greater observed delay.

## Discussion

In this pilot analysis using repurposed bolus tracking monitoring data, contrast arrival in the popliteal artery was delayed in the flexed limb at maximum knee flexion (median +1.5 s), with a smaller, nonsignificant delay at 90-degree flexion. The within-subject increase in arrival delay from 90-degree flexion to maximum flexion (p = 0.018) is consistent with a mechanical contribution to the observed differences, complementing the morphological and computational findings of Nagita et al.,^6)^ who described flexion-induced vessel kinking and an unfavorable computed hemodynamic state in the same anatomical context. Because the flexed limb was, by design, the affected limb, we interpret the between-limb delay primarily as evidence that the bolus tracking-based TEC method can detect a positional difference in contrast arrival. Furthermore, the within-limb, angle-dependent increase—in which each limb acts as its own control—is the finding least confounded by baseline interlimb disease asymmetry. Accordingly, we describe the between-limb finding as an association between the flexed, more severely affected limb and delayed contrast arrival, rather than as evidence that knee flexion itself caused the delay.

Several features of the study design constrain how strongly these observations can be attributed to knee flexion alone. By protocol, the affected limb (ABI ≤0.90) was always assigned to the flexed position, and the contralateral limb served as the extended control; consequently, positional and disease-burden effects cannot be fully separated in the present cohort. The supporting correlations between arrival delay and ABI asymmetry (**Supplementary Fig. S4A**) and between arrival delay and flexed-limb ABI in patients with unilateral PAD (**Supplementary Fig. S4B**) are compatible with both mechanical impairment from flexion and a contribution from baseline interlimb disease asymmetry. The angle-dependent within-subject increase in delay is more difficult to explain by baseline disease alone, but its magnitude (median +0.5 s) approaches the temporal resolution of the acquisition, and we therefore interpret this as suggestive rather than confirmatory evidence of mechanically driven flow impairment. Because the median increase equals 1 sampling interval, its absolute value should not be overinterpreted; our inference rests on the consistency of the direction of change across paired observations rather than on the size of the increment. Quantization at this sampling interval can only shift an observed paired difference toward 0 or toward 1 full interval, so the resulting estimate is more likely to be conservative than exaggerated. This result is therefore best regarded as supportive, hypothesis-generating evidence for a possible mechanical contribution of flexion angle rather than as direct confirmation of flexion-induced flow impairment.

### Dissociation between arrival time and slower TEC parameters

The dissociation between the positive arrival-time finding and the negative findings for rise rate and iAUC merits attention. Contrast arrival time primarily reflects bulk transport delay from the upstream injection site to the measurement plane, and is sensitive to a focal increase in transit resistance, such as that produced by vessel kinking. In contrast, rise rate and iAUC integrate enhancement over a longer interval and are influenced by collateral inflow, post-stenotic perfusion, and inter-patient differences in cardiac output and contrast delivery, which we addressed only partially by expressing these parameters as flexed/extended ratios. It is therefore plausible that arrival time is the most sensitive of the 3 parameters to a transient, position-dependent transit delay, while the slower, integrated parameters are dominated by inter-patient variability in the present sample. Larger cohorts with concurrent measurement of systemic hemodynamic variables will be needed to clarify whether rise rate and iAUC carry information incremental to arrival time. Taken together, these considerations indicate that the principal methodological contribution of the present approach is the detection of a difference in contrast arrival, not a comprehensive characterization of lower-extremity hemodynamics; the negative secondary findings should not be read as establishing the absence of positional differences in these parameters.

### Unilateral vs. bilateral PAD subgroups

The contrasting findings between the unilateral and bilateral PAD subgroups are exploratory and can be interpreted in more than 1 way. The simplest interpretation—that the contralateral, non-PAD limb in patients with unilateral PAD provides a relatively normal internal reference, whereas the diseased contralateral limb in patients with bilateral PAD does not—is consistent with the directionality of the observed differences. However, alternative explanations cannot be excluded. First, in bilateral PAD, the “control” limb itself has baseline inflow impairment and may also undergo positional changes (for example, subtle hip-flexion–related effects in the extended limb), thereby narrowing any detectable interlimb difference. Second, patients with bilateral PAD may represent a more advanced systemic phenotype, with reduced cardiac output and altered global contrast kinetics blunting both limbs’ arrival times symmetrically. Third, the bilateral subgroup was small (n = 7), the analysis was underpowered to detect small interlimb differences, and no adjustment for multiple comparisons was applied; these subgroup findings should therefore be regarded as hypothesis-generating. Disentangling these explanations will require a within-limb repeated-measures design with healthy controls and detailed anatomical characterization.

### Pathophysiological framing

If contrast arrival is delayed while the knee is flexed, inflow to the calf may be transiently reduced in postures that occur frequently in daily life, such as kneeling, squatting, seiza, and prolonged sitting with the knees flexed. In patients with PAD, in whom flow reserve is already limited, such a positional reduction could, in principle, contribute to position-related symptoms and to variability in perfusion measurements obtained in different postures. The pathophysiological framework offered by these observations is necessarily preliminary. Contrast arrival delay is a surrogate imaging parameter, and the present study did not assess claudication symptoms, walking distance, tissue perfusion, or functional status. The magnitudes observed (median +1.5 s at maximum flexion) are small in absolute terms and approach the 0.5-s sampling interval of the acquisition. We therefore refrain from inferring a direct relationship between arrival delay and patient-reported symptoms or clinical outcomes from this dataset. Whether bolus tracking–derived TEC parameters provide information incremental to the resting ABI,^27)^ and whether positional flow changes detected by this method correspond to symptom-relevant flow impairment, are questions that future prospective studies with concurrent symptom, perfusion, and functional assessments will need to address. Throughout this report, we have therefore sought to keep the imaging observation separate from its possible clinical significance: what we describe is a measurable temporal difference on monitoring images obtained in 2 positions, not a demonstrated ischemic burden.

### Methodological considerations

A methodological strength of this study is the repurposing of bolus tracking monitoring data—information routinely acquired during CT angiography but typically discarded after serving its timing function. The 0.5-s sampling interval is coarser than that of dedicated dynamic CT perfusion protocols; nevertheless, the median +1.5-s delay observed at maximum flexion corresponds to 3 sampling intervals and is therefore well above the 0.5-s quantization step of the measurement. The fixed arrival-time threshold (baseline + 20 HU, with a 3-consecutive-point requirement applied to the raw data) was applied uniformly to all datasets and calibrated to the cohort’s baseline noise, with the multi-frame requirement and visual confirmation guarding against false detection from frame-to-frame noise. The nonsignificant finding at 90-degree flexion (median +1.0 s, 2 sampling intervals) should be interpreted with this resolution constraint in mind. Unlike Doppler ultrasound, the present approach requires contrast medium administration and ionizing radiation, although these are inherent to the CT angiographic examination from which the data are derived; the TEC analysis itself incurs no additional radiation, contrast medium, or scan time beyond that examination. This economy distinguishes the approach from dedicated dynamic CT perfusion, which samples the entire first-pass curve and is designed for quantification, but requires a dedicated acquisition and a substantially higher radiation dose. Bolus tracking monitoring is acquired at a single fixed table position with low-dose, nondiagnostic settings and terminates as soon as the triggering threshold is reached, so that its duration is variable and its end point is determined by the trigger rather than by the physiology under observation; the coarse temporal sampling, the truncation of the curve, and the censoring of late-arriving observations all follow from this design. It should be emphasized that this economy applies to the analysis rather than to the 2-position imaging protocol from which the present data derive, which was itself undertaken for research purposes.

### Limitations

This study has several limitations. First, and most importantly, the affected limb was consistently assigned to the flexed position by protocol, with the contralateral limb serving as the extended control. As a result, positional effects and baseline interlimb disease asymmetry are inherently confounded, and the present design cannot establish that the observed delays were caused by knee flexion rather than by baseline disease burden in the flexed limb. A within-limb repeated-measures design (the same limb imaged in extension and flexion) would be methodologically stronger and is the preferred approach for future studies. The within-limb angle-dependent analysis partially mitigates this confounding, because each limb’s disease burden is held constant across the 2 flexion angles; however, it does not fully resolve the issue, given the fixed acquisition order, the unstandardized hip position, and the small (median +0.5 s) magnitude of the angle-dependent change. Second, no healthy control subjects were included; therefore, we cannot determine whether the observed positional changes are disease-specific or in part a normal physiological response to knee flexion. Third, anatomical characterization of PAD severity in the present cohort was limited to ABI, without systematic reporting of lesion location, chronic total occlusion, stenosis severity, Trans-Atlantic Inter-Society Consensus (TASC) classification, runoff status, collateral circulation, or popliteal artery involvement. Because contrast arrival time is influenced by upstream inflow disease and arterial anatomy, this limits the specificity of arrival delay as a marker of locally flexion-induced flow impairment.

Fourth, systemic hemodynamic factors known to influence contrast transit (heart rate, cardiac rhythm, cardiac output, ventricular function, and blood pressure) were not measured or adjusted for, and may contribute to inter-patient variability, particularly in the secondary endpoints. Fifth, 8 of the 30 original patients were excluded, mainly because of calcification and beam-hardening artifacts. Because severe calcification is common in advanced PAD, this may bias the analyzed sample toward cases with relatively preserved lumen visualization. Sixth, the imaging order was fixed (90-degree flexion before the maximum flexion) and the hip flexion angle was neither standardized nor measured, both of which may contribute to order or positional confounding effects, although the 5-minute interval between acquisitions was intended to allow contrast washout. We also did not systematically evaluate for popliteal artery entrapment syndrome,^28,29)^ which could contribute to positional flow changes in some patients. Finally, “maximum flexion” refers to the achievable angle for each patient (mean 121.8° ± 13.0°) rather than a physiological maximum, reflecting patient tolerance under the supine imaging protocol. Because the achieved angle varied between patients, the angle-dependent finding describes a within-patient change from 1 position to another rather than a change across a fixed angular increment, and the magnitude of the mechanical stimulus therefore differed between patients.

## Conclusion

In this pilot analysis, knee flexion in patients with PAD was associated with delayed contrast arrival in the popliteal artery of the flexed limb, with a greater effect at maximum flexion than at 90-degree flexion. Because the present design could not separate positional effects from baseline interlimb disease asymmetry, these results are best interpreted as preliminary evidence supporting the feasibility of TEC analysis from bolus tracking data for assessing positional contrast arrival, rather than as definitive evidence of flexion-induced flow impairment. The within-limb increase in delay at maximum flexion compared with 90-degree flexion, although small, was the observation least explained by baseline interlimb differences and provides the strongest internal support for a mechanical contribution. Within-limb repeated-measures studies in anatomically characterized cohorts, ideally with concurrent symptom and perfusion assessments, are required to establish the clinical relevance of this approach.

## Supplementary Materials

Supplementary Methods Threshold determination for arrival time. Analysis of baseline noise across all 88 time–enhancement curve datasets, providing the basis for the baseline + 20 HU arrival threshold and for the requirement of 3 consecutive suprathreshold time points.

Supplementary Fig. S1 Determination of the arrival-time detection threshold from baseline noise. (**A**) Distribution of point-to-point CT value differences during the baseline phase across all 88 datasets. (**B**) Per-dataset baseline noise standard deviation in relation to the 20-HU detection threshold.

Supplementary Fig. S2 Representative time–enhancement curves from a patient with peripheral arterial disease at 90-degree flexion and maximum knee flexion, showing the arrival time of each limb and the arrival delay at maximum flexion.

Supplementary Fig. S3 Distribution of the secondary time–enhancement curve parameters: the rise rate ratio and the initial area under the curve ratio (flexed/extended) at 90-degree flexion and maximum flexion.

Supplementary Fig. S4 Correlations between disease-severity measures and arrival delay. (**A**) Ankle–brachial index difference between limbs vs. arrival delay at 90-degree flexion in all patients. (**B**) Flexed-limb ankle–brachial index vs. arrival delay in the unilateral peripheral arterial disease subgroup at maximum flexion.

## Declarations

### Data availability

De-identified time–enhancement curve parameters, ankle–brachial index values, knee flexion angles, and basic demographic and clinical characteristics are available from the corresponding author (Masayuki Tokunaga) on reasonable request, subject to institutional approval and a signed data use agreement.
