# Supplementary material for: Knee Flexion and Popliteal Artery Contrast Arrival in Peripheral Arterial Disease: A Pilot Analysis of Bolus Tracking Monitoring Data
Source: Ann Vasc Dis. 2026 Oct 20;19(1):26-00095. doi: 10.3400/avd.oa.26-00095 (PMC13690619; doi:10.3400/avd.oa.26-00095)
Supplement: Supplementary file 1 [file avd-19-1-26-00095-s001.pdf]

Supplementary Methods Threshold determination for arrival time.

The 20 HU threshold for arrival time detection was determined through systematic analysis of baseline noise characteristics. For each TEC dataset (n=88), baseline standard deviation was calculated from the initial pre-enhancement phase (first 3 time points). Across all datasets, the mean baseline noise was 7.0 HU (range 0.5-20.1 HU; median 6.8 HU). The 20 HU threshold corresponds to approximately 2.9 standard deviations above the mean baseline noise.

To minimize false-positive arrival detection, we required three consecutive time points exceeding the threshold. Given the approximately normal distribution of point-to-point variations (mean difference ≈ 0 HU; SD = 12.0 HU), the probability of three consecutive false-positive points is negligible, providing robust protection against noise-induced artifacts. Visual inspection of all TEC curves confirmed appropriate arrival detection in all cases.
