# Supplementary material for: Knee Flexion and Popliteal Artery Contrast Arrival in Peripheral Arterial Disease: A Pilot Analysis of Bolus Tracking Monitoring Data
Source: Ann Vasc Dis. 2026 Oct 20;19(1):26-00095. doi: 10.3400/avd.oa.26-00095 (PMC13690619; doi:10.3400/avd.oa.26-00095)
Supplement: Supplementary file 2 [file avd-19-1-26-00095-s002.pdf]

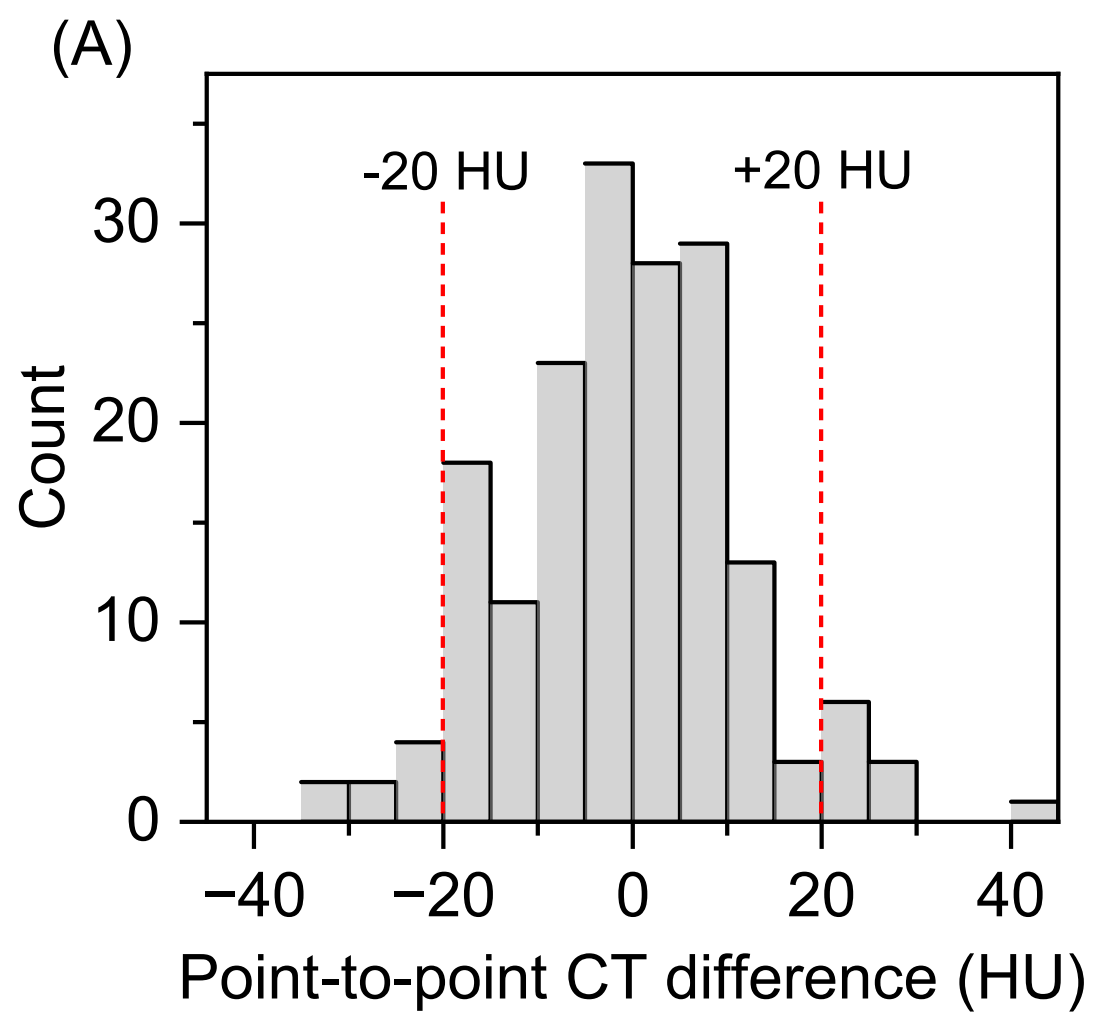

Supplementary Fig. S1 Determination of the arrival-time detection threshold from baseline noise.

(A, B) Threshold determination based on baseline noise analysis. (A) Histogram showing distribution of point-to-point CT value differences during baseline phase (first three time points) across all 88 datasets (mean ≈ 0). Red vertical lines indicate ±20 HU threshold boundaries; the majority of differences fall within this range. (B) Per-patient baseline noise standard deviation. Horizontal red line indicates the 20 HU detection threshold. Baseline SD was below 20 HU in the large majority of datasets (mean 7.0 HU); the requirement for three consecutive supra-threshold time points, together with visual confirmation, guarded against false detection in the few datasets with higher baseline noise.

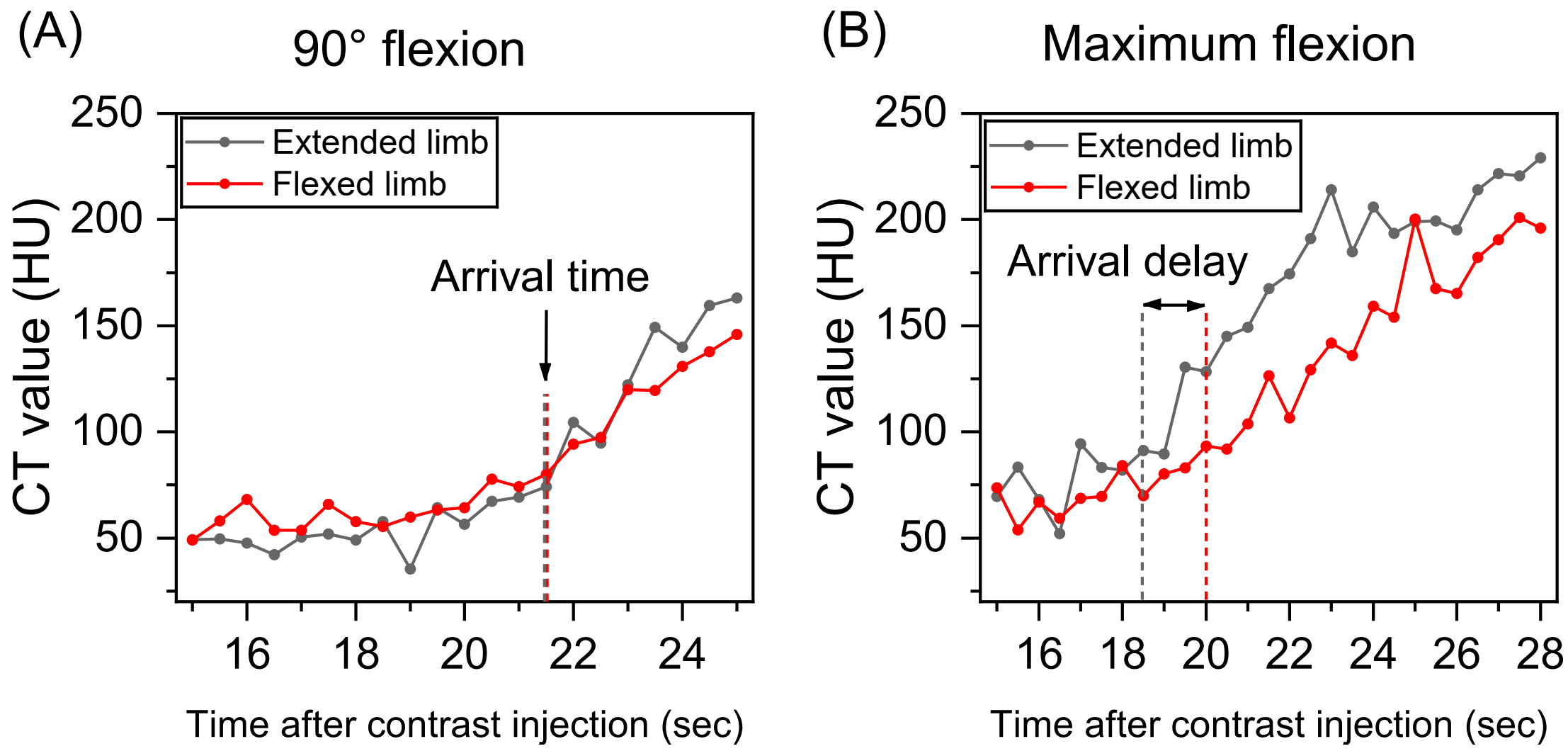

Supplementary Fig. S2 Representative time-enhancement curves (TECs) from a PAD patient (extended limb ABI 1.10; flexed limb ABI 0.81) at two knee flexion angles. (A) At 90-degree flexion, both limbs show nearly simultaneous contrast arrival. (B) At maximum flexion, the flexed limb (red) demonstrates delayed contrast arrival compared to the extended limb (gray), with an arrival delay of 1.5 seconds—consistent with the cohort median. Dots indicate raw CT attenuation values extracted from bolus tracking monitoring images at 0.5-second intervals. Vertical dashed lines mark the arrival time for each limb, defined as the first of three consecutive time points exceeding baseline + 20 HU.

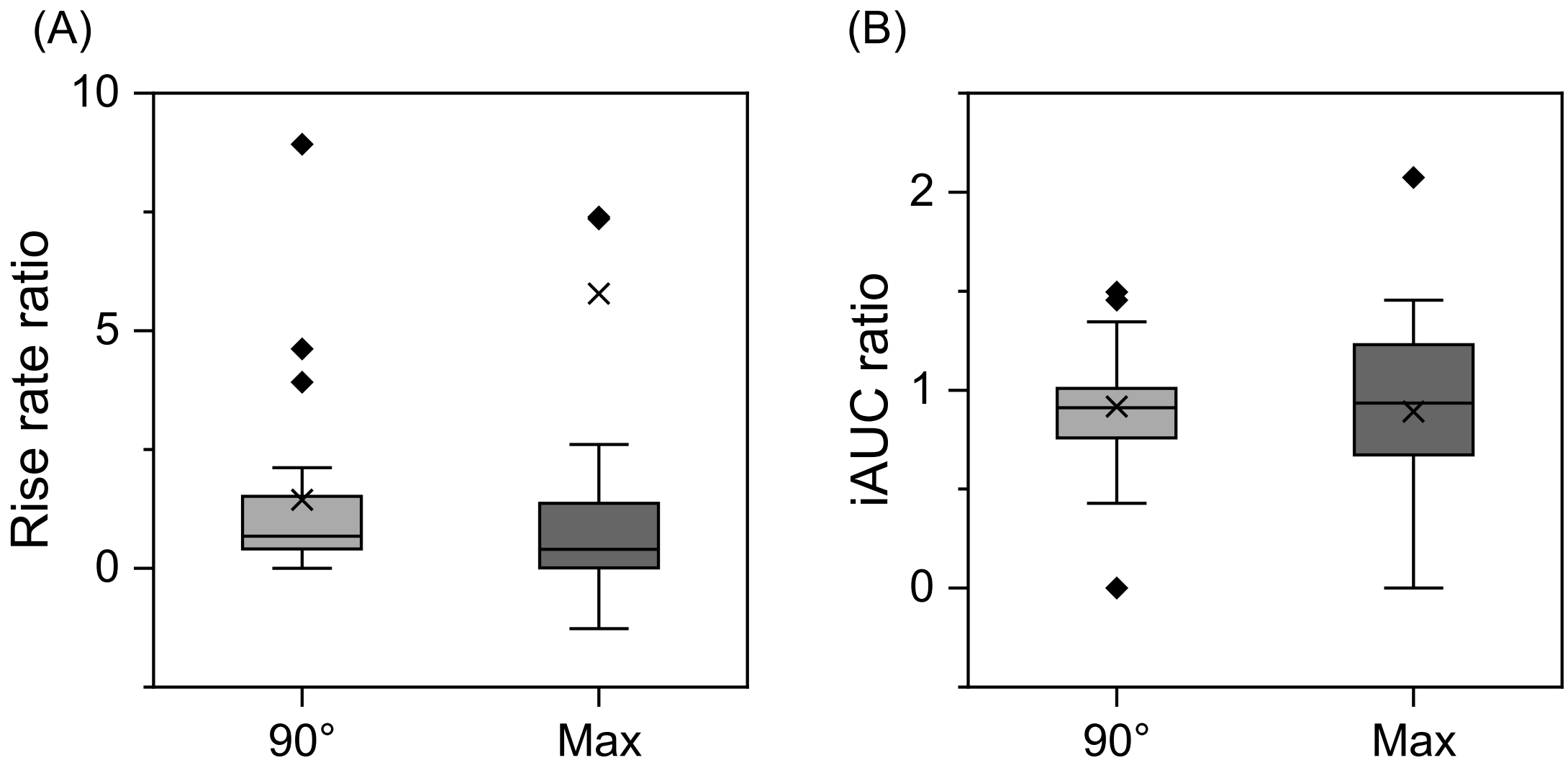

Supplementary Fig. S3 Distribution of secondary TEC parameters. (A) Rise rate ratio (flexed/extended) at 90-degree and maximum flexion. One extreme outlier (102.7) at maximum flexion exceeds the displayed range. (B) iAUC ratio distribution showing more moderate variability.

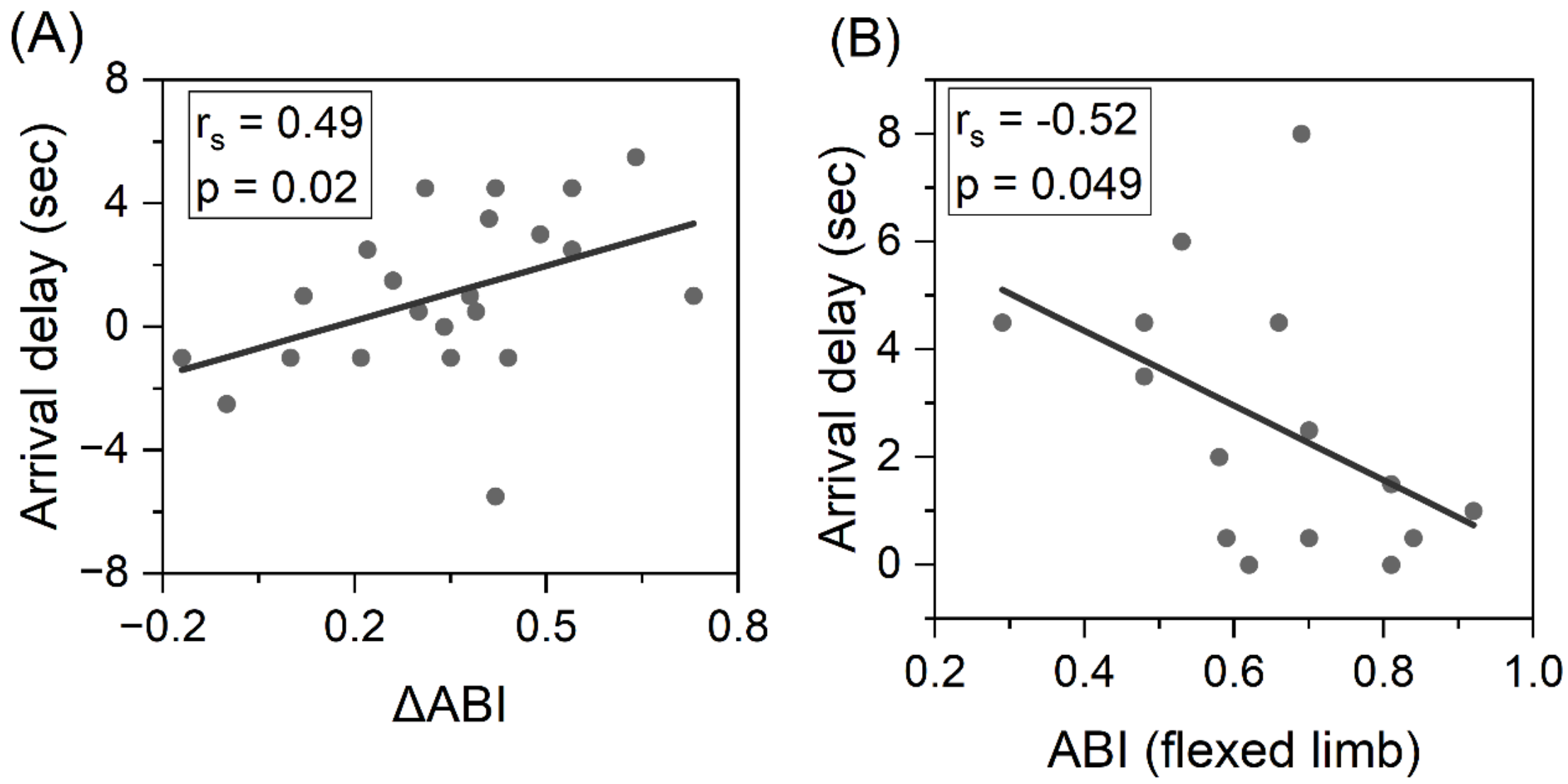

Supplementary Fig. S4 Correlations between disease-severity measures and arrival delay. (A) ABI difference (delta-ABI = extended-limb ABI minus flexed-limb ABI; positive values indicate more severe disease in the flexed limb) versus arrival delay (flexed minus extended limb) at 90-degree flexion, all patients; larger ABI difference was associated with greater arrival delay (Spearman rho = 0.49, p = 0.020). (B) Flexed-limb ABI versus arrival delay in the unilateral PAD subgroup (n = 15) at maximum flexion; lower ABI (more severe disease) was associated with greater arrival delay (Spearman rho = -0.52, p = 0.049). Solid lines indicate linear regression fits for visualization.
